# An Efficient Short-Term Traffic Speed Prediction Model Based on Improved TCN and GCN

**DOI:** 10.3390/s21206735

**Published:** 2021-10-11

**Authors:** Zhiqiu Hu, Rencheng Sun, Fengjing Shao, Yi Sui

**Affiliations:** 1 School of Automation, Qingdao University, Qingdao 266071, China; huzhiqiu@qdu.edu.cn; 2 College of Computer Science and Technology, Qingdao University, Qingdao 266071, China; sfj@qdu.edu.cn (F.S.); freesui1984@163.com (Y.S.)

**Keywords:** temporal convolutional network, 2D dilated convolution, graph convolution network, spatial-temporal correlation, short-term traffic speed prediction

## Abstract

Timely and accurate traffic speed predictions are an important part of the Intelligent Transportation System (ITS), which provides data support for traffic control and guidance. The speed evolution process is closely related to the topological structure of the road networks and has complex temporal and spatial dependence, in addition to being affected by various external factors. In this study, we propose a new Speed Prediction of Traffic Model Network (SPTMN). The model is largely based on a Temporal Convolution Network (TCN) and a Graph Convolution Network (GCN). The improved TCN is used to complete the extraction of time dimension and local spatial dimension features, and the topological relationship between road nodes is extracted by GCN, to accomplish global spatial dimension feature extraction. Finally, both spatial and temporal features are combined with road parameters to achieve accurate short-term traffic speed predictions. The experimental results show that the SPTMN model obtains the best performance under various road conditions, and compared with eight baseline methods, the prediction error is reduced by at least 8%. Moreover, the SPTMN model has high effectiveness and stability.

## 1. Introduction

Transportation is one of the most important components in society. It is the foundation for a country to develop healthily, rapidly, and sustainably. As a result of the development of the social economy, traditional traffic management technology struggles to deal with the ever-increasing traffic pressure, and there is an urgent need to develop Intelligent Transportation Systems (ITS), of which traffic prediction is an important part. Scholars have conducted a large amount of research in this field [1,2]. As one of the three key traffic parameters, average road speed is an important tool to reflect the traffic state and has gradually become one of the major constituents of current traffic prediction. Short-term traffic speed prediction can use real-time traffic data to predict the traffic conditions within the next few minutes, and accurate and timely predictions play a key role in traffic condition improvement, traffic event prediction, and traffic travel organization [3].

There is a complex spatial-temporal correlation relating to short-term traffic speed. In the time dimension, there is a correlation between the current traffic state and the traffic state in the recent or extended past. To demonstrate the time correlation, an Autocorrelation Function (ACF) [4] was introduced. In the ACF, a column of data is divided into two columns according to a lag number, and the correlation coefficient of the two columns of data is then calculated.

The formula is:

(1) $$\overset{n-t}{\underset{i=1}{\sum }}\frac{(x_{i}-u)\left(x_{i+t}-u\right)}{\sum_{i=1}^{n}{\left(x_{i}-u\right)}^{2}}$$

**Figure 1:**
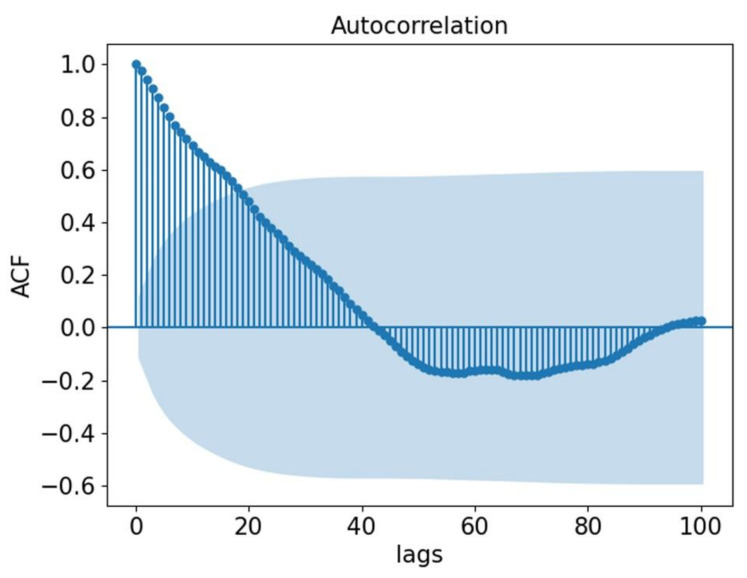
Temporal correlation of traffic speed.

Figure 1 shows the ACF of randomly selected traffic speed time series data, and the interval between each time point is 5 min. The confidence interval is set to 95%, which is the blue shaded area. It is obvious that the current time is highly autocorrelated with the first-order lag time, and the ACF value before lag time 19 is above the confidence interval; that is, there is autocorrelation between the two time points. However, it can also be seen that the ACF gradually decreases with the increase in the number of lags.

**Figure 2:**
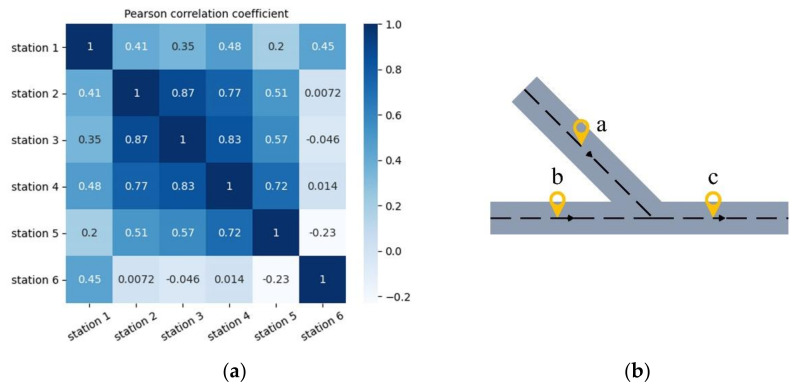
Spatial correlation of traffic speed. (**a**) Thermodynamic diagram of the Pearson coefficient between traffic speeds. (**b**) Spatial correlation between traffic speeds on different roads.

Sections of the road network are interconnected according to a certain topological structure. The traffic flows distributed on different road sections affect each other, which forms the spatial correlation of the traffic flow. This correlation exists not only between the upstream and downstream sections, but also between different roads. Figure 2a shows the Pearson coefficient of traffic speed between six adjacent sensor stations on the same expressway. The figure illustrates that spatial dependence is related to the distance between sections. The smaller the distance, the greater the correlation coefficient, and conversely, the correlation coefficient decreases with an increase in distance. In Figure 2b, section A is not on the same road as sections B and C, and the traffic flows from sections A and B downstream to section C. When the traffic flow of section A increases, it inevitably increases the flow within section C and causes traffic stagnation. The stagnation of section C will also affect the upstream direction in section B and slow the vehicle speed in section B accordingly. Additionally, speed is affected by many other factors, including geographical location, traffic time changes, and the number of lanes open. These external factors also lead to the instability and non-linearity of traffic speed, which makes the task of traffic speed prediction extremely challenging.

As the basic component of traffic control and guidance, short-term traffic speed prediction has always been an area of interest for study. Existing traffic speed prediction models are generally based on statistical models and traditional machine learning algorithms, such as the Auto-Regressive Integrated Moving Average model (ARIMA) [5], Kalman filter algorithm (KF) [6,7], and Support Vector Machine (SVM) [8], but ARIMA and KF struggle to process unstable and nonlinear data. The information mining ability of SVM is stronger than that of the first two methods, but it has a single structure and lacks the ability to extract deeper features, and cannot deal with excessive amounts of data.

As a branch of machine learning, deep learning has risen rapidly in recent years and promoted the development of artificial intelligence. It has permitted some outstanding achievements in various applications such as computer vision, speech recognition, and natural language processing [9,10,11]. In recent years, the deep learning model, represented by Recurrent Neural Networks (RNN) and Convolutional Neural Networks (CNN), has been increasingly used in the study of traffic prediction. RNN has a cyclic auto-regressive structure, so RNN and its variants Long Short-Term Memory (LSTM) and Gate Recurrent Unit (GRU) are the most frequently used in the field of time series modeling. Colin, Lea et al. proposed a Temporal Convolutional Network (TCN) in 2016, which was specifically designed for time series data modeling and behavior recognition and detection applications [12]. In contrast to the RNN structure, TCN can be processed in a large-scale parallel mode, so it will run faster during training and verification. Moreover, TCN can change the receptive field by increasing the number of layers, changing the dilation rate and kernel, which makes the length of input data more flexible, and avoids the problems of gradient dispersion and gradient explosion [13]. Compared with a number of RNN variants, TCN can reach or even exceed their performance in a variety of tasks.

CNN is recognized as the main force in the field of image and video recognition. Although it can extract spatial dependence, CNN is only applicable to European space data, such as images, video, etc. The traffic sensor network is complex and irregular, and CNN cannot effectively characterize the spatial dependence. To deal with irregularly structured data, the Graph Convolution Neural Network (GCN) was devised. GCN is an extension of CNN in the graph domain. It can learn node features and structural features at the same time and it is currently the best choice for graph data learning tasks. Considering the above factors, this paper proposes a new end-to-end spatial-temporal graph neural network model (SPTMN) for short-term speed prediction. The model can effectively process the spatial-temporal features and the geographical structure characteristics of roads. The main accomplishments of this study are as follows:

- According to the temporal and spatial characteristics of traffic speed data, we replaced the one-dimensional dilated convolution in TCN with the two-dimensional dilated convolution to form a two-dimensional Temporal Convolutional Network (2DTCN), which can extract the local spatial features whilst also extracting the temporal features.
- In GCN, we added the distance information between nodes to the adjacency matrix to form a weighted adjacency matrix, to extract the global spatial correlation between nodes and improve the prediction accuracy of the model.
- We developed the SPTMN model by combining 2DTCN and GCN. The model uses 2DTCN to achieve feature enhancement and extract temporal and local spatial features, and uses GCN to obtain the topology between road nodes, to model the spatial dependence. Finally, the geographical structure features of the road are integrated into the model.
- We evaluated the proposed model on real traffic speed datasets. The experimental results show that compared with the existing baseline models, the SPTMN model obtains the best prediction performance, and the SPTMN model is more stable and efficient.

The remainder of this paper is organized as follows: Section 2 briefly reviews the research related to speed prediction. Section 3 introduces the problem definition and SPTMN framework structure. Section 4 evaluates the prediction performance of SPTMN model, including prediction results comparison and analysis, case study, and interpretation study. Finally, Section 5 presents the conclusions and further research.

## 2. Related Research

Short-term traffic speed prediction refers to predicting the traffic speed in the next period according to the real-time and historical traffic speed. Because traffic speed prediction plays an important role in intelligent transportation, researchers have tried many methods to establish prediction models. These methods can be divided into three categories: the statistical theory method, the traditional machine learning method, and the deep learning method.

The traffic prediction model based on statistical theory mainly fulfills a single-point prediction of a univariate time series. The most used are ARIMA and KF. ARIMA assumes that traffic is a stationary process with invariant mean, variance, and auto-correlation. Levin and Taso used ARIMA to predict traffic flow for the first time, and the prediction results were superior to other ad hoc smoothing models [14]. The ARIMA model was also applied to rail transit passenger flow prediction [15]. The Kalman filter uses the linear system state equation to estimate the optimal system state. Zhou et al. predicted the average future speed of road sections by using the average speed of vehicles upstream and downstream based on the KF model [16]. Traditional statistical methods lack the ability to deal with nonlinear relationships and high-dimensional data, so it is difficult to ensure the accuracy and reliability of traffic speed prediction based on statistical models in highly nonlinear and stochastic traffic systems.

Within the past two decades, machine learning has been successfully applied in many fields, and researchers have also applied it to traffic prediction modeling. Classic machine learning methods include Support Vector Machine (SVM) [17], Bayesian Network (BN) [18], and K-Nearest Neighbors (KNN) [19]. However, these learning methods need to manually extract traffic features, and the method of selecting features often changes with the change in the conditions, so they cannot extract the spatial correlation between traffic data and consequently cannot get reliable prediction results in complex traffic prediction tasks.

In recent years, as a result of traffic sensor system improvements, the quantity of traffic flow data has increased rapidly. Using extensive amounts of traffic data, researchers have applied deep learning models to traffic prediction. The deep learning method can automatically mine the hidden features of traffic data and better deal with increasingly higher spatial and temporal dimensions. The classic deep learning models, LSTM and CNN were the first to be applied in traffic speed prediction. Yu et al. [20] used deep belief network (DBN) and LSTM models to consider rainfall data to predict the speed of main roads in Beijing. The results showed that the LSTM predictions were better than that of DBN. Aniekan et al. [21] proposed a bi-directional Long Short-Term Memory Neural Network (LSTM-NN) model and combined it with weather data to predict the short-term traffic speed of the main road through Manchester. Wu et al. [22] combined LSTM with an attention mechanism to create the ATT-LSTM model and applied it to road traffic speed prediction in Shenzhen. Results showed that the attention mechanism greatly improved prediction accuracy. The methods mentioned above can only extract the temporal dependence of the traffic speed of a single road section or a single sensor, without considering spatial dependence. Therefore, several studies have attempted to extract spatial correlation using CNN. The key obstacle to this research is how to input spatial dependence data into CNN. Wang C. [23] integrated the speed data of adjacent sections into a two-dimensional matrix and input it into an Error-feedback Recurrent Convolutional Neural Network (eRCNN) and predicted the taxi speeds on the Beijing ring road. Ma et al. [24] used a two-dimensional space-time matrix to transform the traffic state into an image and used CNN to extract the traffic characteristics in the image and predict traffic speed. Inspired by this, in [25], a grid representation method was proposed which can preserve the fine structure of the network. The traffic image was modeled as a video with time as a variable, and then the Spatiotemporal Recurrent Convolutional Networks (SRCNs) composed of DCNNs and LSTM was used to extract the temporal and spatial dependence of the data. Yao et al. [26] proposed the Spatial-Temporal Dynamic Network (STDN), which used a flow-gating mechanism and CNN to extract spatial data correlation, and used the attention mechanism and LSTM to extract temporal correlation.

Traditional convolution is limited to processing Euclidean data such as images, which is not sophisticated enough for the complex topology of traffic data, and a graph data structure can satisfactorily characterize this structure. Therefore, many studies have used GCN to model spatial dependence. Zhao et al. [27] combined GCN with GRU to propose the T-GCN model and applied it to traffic prediction. Results showed that the T-GCN could adequately extract the spatio-temporal features of data. Yu et al. [28] combined GCN and gated temporal convolution to establish the Spatio-Temporal Graph Convolutional Network (STGCN), which is composed solely of the convolutional neural network. The model had fewer parameters and could effectively be applied to large-scale datasets. On this basis, Feng et al. [29] proposed the MSTGCN model and applied it to expressway flow prediction. MSTGCN consisted of three independent components to model the recent, daily, and weekly dependence of traffic data, respectively. Each component was composed of GCN and standard two-dimensional convolution. The Attention-Based Spatial-Temporal Graph Convolutional Network (ASTGCN) was based on MSTGCN with a spatial-temporal attention mechanism [30]. Although this GCN-based model can extract the temporal and spatial characteristics of traffic data, it does not take into account other traffic auxiliary information, such as the geographical location of monitoring points, traffic flow direction, and number of lanes. For example, the morning and evening peak duration and speed change range of monitoring stations with different distances from the city center are different. Due to the above shortcomings, this study proposes the SPTMN model to model the temporal and spatial characteristics of traffic data based on the consideration of traffic auxiliary information.

## 3. Methodology

### 3.1. Problem Definition

Short-term traffic speed prediction uses the historical data of the first *t* time points of *N* traffic sensors to predict the traffic speed values of these *N* sensors at *d* time points in the future, to provide support for traffic management and control. The evolving traffic state is closely related to the road network structure; therefore, the representation of the relationship between roads, and the setting of relationship weight, are important factors that affect the accuracy of traffic speed prediction. As the traffic speed data is obtained through sensors deployed on the road network, the sensor network is used to mimic the road network. We used undirected graph *G* (*V*, *E*, *W*) to represent the sensor network, where $\left|V\right|=N$ is the vertex set, which represents *N* sensors, and *E* is the edge set. If there are two adjacent sensors in the road network, there is an edge between them and $W\in R^{N\times N}$ is the relationship weighting between *N* vertices. It is a symmetrical matrix, which is obtained by calculating the distance between the two sensors through the sensor coordinates.

**Figure 3:**
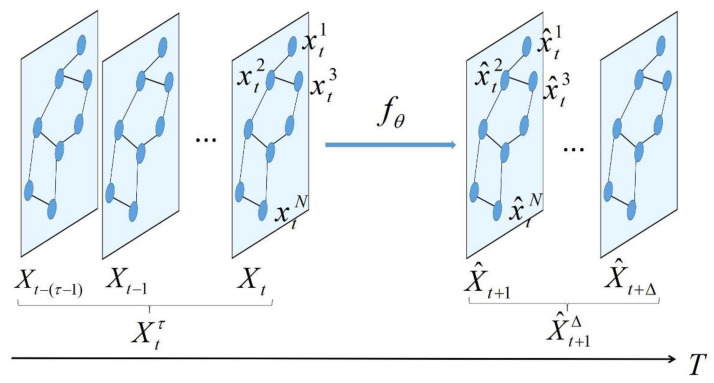
Speed prediction problem definition. The blue node represents the sensor, and the graph structure represents the sensor network.

Each vertex obtains the traffic speed data collected by the detector with frequency F.$x_{t}^{}$ represents the speed value collected by vertex *v* at time *t*. $X_{t}=\left(x_{t}^{1},x_{t}^{2}\ldots ,x_{t}^{N}\right)\in R^{N}$ represents the *n*-dimensional vector composed of *N* speed values monitored by *n* vertices at time *t*. $X_{t}^{\tau }={\left(X_{t-(\tau -1)},X_{t-(\tau -2)},X_{t-(\tau -3)},\ldots ,X_{t}\right)}^{T}\in R^{\tau \times N}$ is the speed value monitored by *N* nodes from time t−(τ−1) to time *t*. ${\hat{X}}_{t+1}^{\Delta }={\left({\hat{X}}_{t+1},{\hat{X}}_{t+2},{\hat{X}}_{t+3},\ldots ,{\hat{X}}_{t+\Delta }\right)}^{T}\in R^{\Delta \times N}$, ${\hat{X}}_{t+1}=({\hat{x}}_{t+1}^{1},{\hat{x}}_{t+1}^{2},\ldots ,{\hat{x}}_{t+1}^{N})$ is the predicted speed of *N* nodes at the time t+1 and ${\hat{X}}_{t+1}^{\Delta }$ is the predicted speed of all nodes of Δ step from time t+1, as shown in Figure 3.

The model also uses traffic auxiliary information as parameters, so $p_{n}^{i}$ is defined as the i-th parameter of the *n*-th node, $p_{n}=(p_{n}^{1},p_{n}^{2},\ldots ,p_{n}^{c})$ represents all the parameters of the *n*-th node, and the parameter matrix $P={\left(p_{1},p_{2},\ldots ,p_{N}\right)}^{T}\in R^{N\times c}$ represents all the parameters of the *n*-th node. Therefore, the short-term traffic speed prediction can be expressed by function f, and the mapping relationship of the function is:

(2) $${\hat{X}}_{t+1}^{\Delta }=f_{\theta }(X_{t}^{\tau };P;G)$$

where θ is the parameter learned by the model in the prediction process.

### 3.2. SPTMN Model Framework

**Figure 4:**
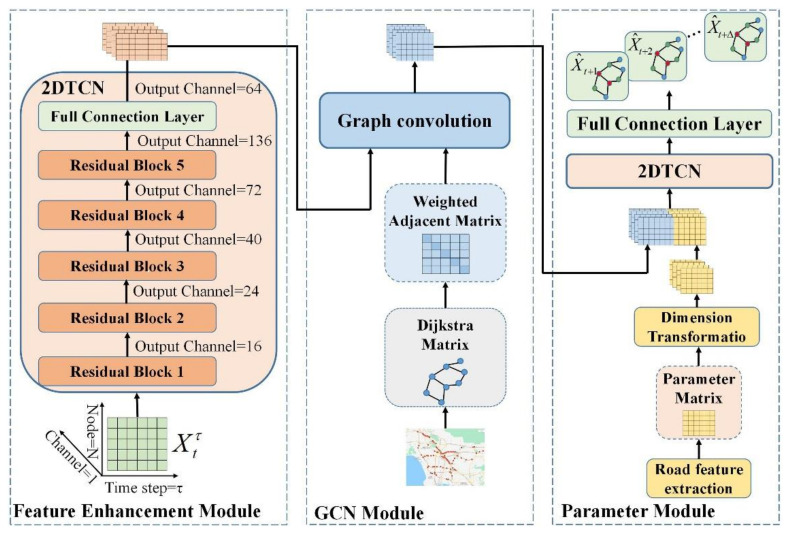
SPTMN model framework.

This section explains the SPTMN model framework in detail. As shown in Figure 4, the SPTMN model consists of three parts. The first part is the feature enhancement module. The input data of this module is the time series data obtained by the sensor station. The input channel is 1, i.e., speed and the feature is single. Therefore, 2DTCN expands the feature dimension of the data to 64 through 5-layer residual blocks to realize feature enhancement and preliminary spatio-temporal feature extraction. The second part is the graph convolution module. In this module, the weighted adjacency matrix is obtained by the Dijkstra algorithm according to the coordinates and adjacency relationship of monitoring stations, and then GCN extracts the global spatial dependence of data based on the weighted adjacency matrix. The third part is the parameter module, in which the geographic structure information of these roads is extracted to form the parameter matrix, the parameter matrix is fused with the output of graph convolution, and then the high-dimensional spatio-temporal features and parameter dimensional features are extracted by 2DTCN. Finally, the final prediction results are obtained by FCN. The following is a detailed explanation of these three modules.

#### 3.2.1. Feature Enhancement Module

The main idea of the feature enhancement module is to introduce additional neural network modules into the input data, increase the feature vector dimensions, and enrich its data representation ability to improve the prediction performance. The feature enhancement module proposed in this study is completed by 2DTCN.

**Figure 5:**
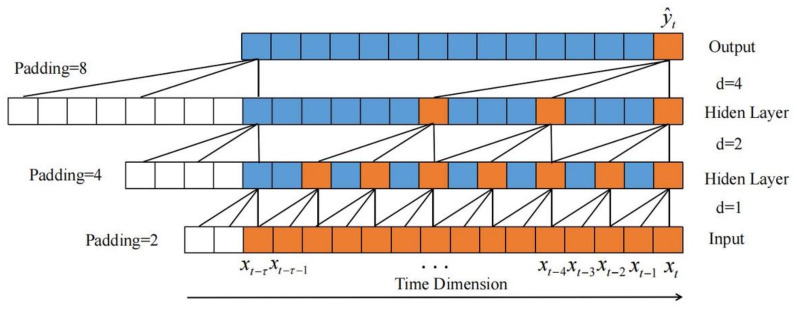
The structure of causal dilated convolution.

TCN consists of causal convolution, dilated convolution, and residual block [31]. Causal convolution means that the output of time t is obtained by convolution of the elements at time t and before, which is just in line with the characteristics of the traffic speed data. The receptive field of causal convolution is linear with the depth of the network. Dilated convolution was introduced to extract longer-term dependencies. Dilated convolution means that holes are injected into kernels, through which a larger receptive field can be obtained, compared with standard convolution. If the kernel is $F=(f_{0},f_{2},\ldots f_{k-1})$ and the input sequence is $\;X\in R^{T\times s}$, then the causal dilated convolution at $x_{t}$ can be expressed as:

(3) $$F_{d}(x_{t})=(F_{d}*X)(x_{t})=\sum_{i=0}^{k-1}f_{i}\cdot x_{t-d\cdot i}$$

where *k* is the size of the kernel and d is the dilated rate. t−d⋅i is the time dimension direction. The dilated rated increases exponentially with the increase in the number of convolutional layers; that is, the dilated rate of the causal dilated convolution of the *i*-th layer is $2^{i-1}$, and the receptive field size of each layer is $(k-1)2^{i-1}+1$. To ensure that the length of each layer’s input and output sequences were the same, padding was added in the process of the convolution. Generally, the padding strategy was to add 0 and the size was (k−1)d. The structure of the 3-layer causal dilated convolution with the kernel of 3 is shown in Figure 5.

To learn the long-term dependence, the network depth needs to be increased, and the complexity of network training will increase accordingly. To reduce complexity and increase stability, the residual block is introduced for deep network training. After adding residual blocks, the output result of the network is:

(4) $$F(x_{T})=F_{d}(x_{T})+x_{T}^{}$$

**Figure 6:**
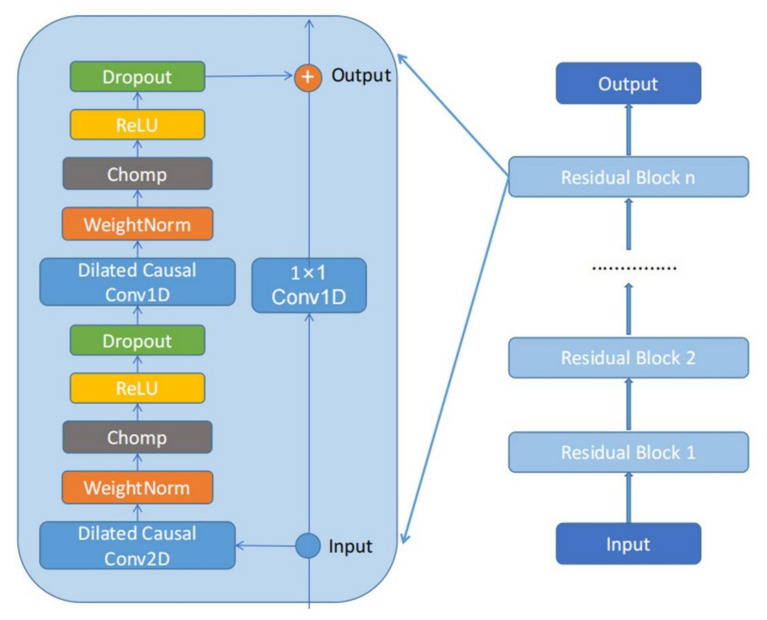
The structure of the residual block of TCN.

The residual block generally consists of two layers of causal dilated convolution, a weight normalization layer, a clipping layer, an activation function, and a dropout layer. The clipping layer can keep the feature-length constant, the activation function uses the Rectified Linear Unit (ReLU), and the dropout layer prevents the network from overfitting. Finally, 1 × 1 convolution changes the dimensions of the input data to be consistent with the output data. TCN usually consists of multiple stacked residual blocks. The TCN residual block structure is shown in Figure 6.

**Figure 7:**
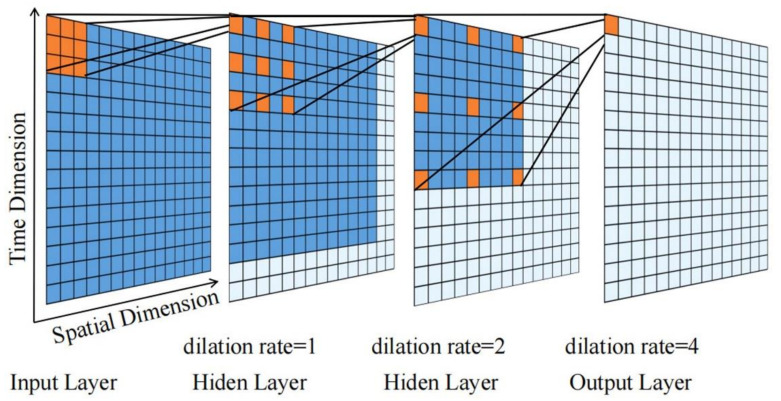
The structure of two-dimensional void convolution, where the blue region is the receptive field of an eigenvalue in the output layer in each layer.

A linear transformation is required before data can be inputted into the graph convolutional network, and a normal linear transformation method will reduce accuracy. Furthermore, the original traffic data at each sensor station has speed as its only dimension and the data is single. Consequently, this paper uses improved TCN to perform linear transformation and feature enhancement on the original data. The original TCN is designed for time series and can only extract the temporal correlation from different time points of the same time series, so the aggregation and correlation amongst traffic nodes is problematic. To initially extract and enhance the spatial-temporal feature of the data at the same time, two-dimensional convolution is used instead of one-dimensional convolution in the causal dilated convolution. The structure of 2DTCN based on 2D convolution is shown in Figure 7. Two-dimensional convolution can not only extract the temporal dimension features of the same node but also extract the spatial dimension features between nodes within the receptive field. If the kernel is $F\in R^{k\times k}$ and the input sequence is $X\in R^{T\times S}$, then the 2D causal dilated convolution at $x_{ts}$ can be expressed as:

(5) $$F_{d}(x_{ts})=(F_{d}*X)(x_{ts})=\sum_{i=0}^{k-1}\sum_{j=0}^{k-1}f_{ij}\cdot x_{\left(t-d\cdot i)(s-d\cdot j\right)}$$

where *d* is the dilated rate, *i* and *j* represent row *i* and column *j* of convolution kernel, respectively, t−d⋅i is the temporal dimension direction, and s−d⋅i is the space dimension direction.

To adapt to the model changes, the 1 × 1 convolution in the residual block has to be changed accordingly. In the convolution process, the input and output data change with the number of channels, and temporal and spatial dimensions. The Chomp function of the clipping layer clips the spatial dimension to keep the number of nodes constant. Therefore, it is necessary to replace 1 × 1 convolution with two-dimensional convolution in the residual block to change the channel number and time dimension of the input data and make it consistent with the output data, to ensure that the two data points can be added by bit. The number of output channels of the two-dimensional convolution is the same as that of the last layer of causal dilated convolution, the kernel is (2×padding+1)×1, and padding is the padding value added by the causal dilated convolution. After 2DTCN, the FCN is added to adjust the data dimension to meet the input requirements of the graph convolution neural network.

#### 3.2.2. Graph Convolution Neural Network Module

Road sensor stations have distinctive topological relationships in different regions, and the interaction between stations with different topological relationships must therefore be different. If the topological relationship between stations can be fully extracted and utilized, the speed prediction will be more accurate [32]. The traditional deep learning method has been very successfully applied to extract the features of regular Euclidean spatial data. However, the topological relationship between road sensor stations is a non-European type graph structure. It is irregular and has no translation invariance. In order to process graph data, previous studies proposed the graph convolution neural network (GCN), which uses the spectral method to extend convolution operation from European data to graph data.

According to the convolution theorem, the convolution operation of two signals can be converted to the frequency domain by Fourier transformation, and then the product operation of the signals in the frequency domain is able to be performed. Finally, the convolution result is obtained by inverse Fourier transformation [33]. The formula is:

(6) $$f*g=F^{-1}(F(f)\cdot F(g))$$

where F and $F^{-1}$ are Fourier transformation and inverse Fourier transformation, f, g are the two signals to be convoluted, ∗ is the convolution operation, and ⋅ is the product operation. The basis of the traditional Fourier transformation is the characteristic function $e^{-i\omega t}$ of the Laplace operator, and the Laplace operator on the graph is the Laplace matrix [33]. Therefore, we need to find the eigenvector of the Laplace matrix corresponding to the graph, so as to find a set of Fourier transformed bases.

The Laplace matrix of a graph L is defined as: L=D−A, where $D\in R^{N\times N}$ is the degree matrix. $A\in R^{N\times N}$ is the adjacency matrix. After standardization, $L=I_{N}-D^{-\frac{1}{2}}AD^{-\frac{1}{2}}$, where $I_{N}$ is the identity matrix. In an undirected graph, D and A are symmetric matrices, so L is a positive semidefinite matrix. $L=U\Lambda U^{T}$ can be obtained by eigenvalue decomposition, where $\Lambda =diag([\lambda_{1},\cdots ,\lambda_{n}])\in R^{N\times N}$, the elements on the diagonal are the eigenvalues of the Laplace matrix, the *i*-th column of $U\in R^{N\times N}$ is the eigenvector corresponding to the eigenvalue $\lambda_{i}$, and U is the orthogonal matrix, that is, the basis of the Fourier transform. Therefore, the convolution formula of the graph can be expressed as:

(7) $$g*x=U(U^{T}g⨀U^{T}x)=Ug_{\theta }U^{T}x$$

where x is the original eigenvector of the node in the graph, $U^{T}$ is the transpose of U, $g_{\theta }$ = $gU^{T}g$ is the learnable kernel, and ⊙ is the Hadamard product.

The complexity of the eigen decomposition of the Laplace matrix is O(n^2^). Defferrard et al. used the Chebyshev polynomial to fit the kernel $g_{\theta }(\Lambda )$ to reduce the computational complexity [34], Hammond et al. pointed out that $g_{\theta }(\Lambda )$ can be fitted by the *k*-order truncation expansion of the Chebyshev polynomial $T_{k}(x)$ [35]. Therefore, the spectral domain graph convolution can be defined as: $g_{\theta }*x=\sum_{i=0}^{K}\theta_{i}T_{k}(\tilde{L})x$, where $\tilde{L}=2L/\lambda_{\max}-I_{N}$, $\lambda_{\max}$ is the maximum eigenvalue of L, $\theta \in R^{K}$ is the coefficient of the Chebyshev polynomial. This expression has local connectivity, and the information of the k-order neighborhood of the central node is extracted every convolution. Kipf et al. introduced a first-order approximation, ChebNet [36], assuming that K=1, $\lambda_{\max=2}$, and each layer of convolution only considers the direct neighborhood. The ChebNet convolution formula is approximately simplified as: $g_{\theta }*x=\theta_{0}x-\theta_{1}D^{-\frac{1}{2}}AD^{-\frac{1}{2}}x$. To reduce the number of parameters and prevent overfitting, assuming $\theta =\theta_{0}=-\theta_{1}$, the first-order approximate model of graph convolution is obtained as follows: $g_{\theta }*x=\theta (I_{N}+D^{-\frac{1}{2}}AD^{-\frac{1}{2}})x$. In order to avoid numerical instability, a self-ring is added to each node of the graph, that is $\tilde{A}=A+I_{N}$, ${\tilde{D}}_{ii}=\sum {}_{j}{\tilde{A}}_{ij}$, to get the formula $g_{\theta }*x=\theta ({\tilde{D}}^{-\frac{1}{2}}\tilde{A}{\tilde{D}}^{-\frac{1}{2}})x$, and finally add the activation function to get the final fast graph convolution formula:

(8) $$H^{\left(l+1\right)}=\sigma ({\tilde{D}}^{-\frac{1}{2}}\tilde{A}{\tilde{D}}^{-\frac{1}{2}}H^{\left(l\right)}W^{\left(l\right)})$$

where $H^{\left(l\right)}\in R^{N\times C}$ is the input feature of layer l, C is the feature dimension of each node, $W^{\left(l\right)}\in R^{C\times M}$ is the weight matrix, M is the number of output channels, $H^{\left(l+1\right)}\in R^{N\times M}$ is the output feature of layer l, and σ is a nonlinear activation function.

**Figure 8:**
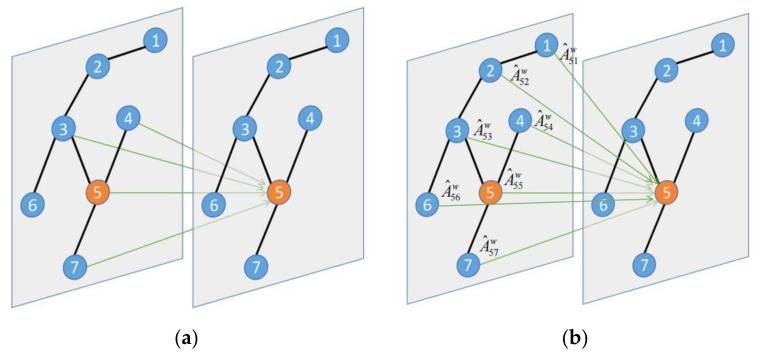
Principle of graph convolution neural network. (**a**) Graph convolution neural network based on adjacency matrix. (**b**) Graph convolution neural network based on the distance matrix.

As shown in Figure 8a, the essence of GCN is actually a linear combination of neighbor features and its own individual node features. A multi-level GCN will lead to an over-smoothing problem [37], which makes node discrimination consistently worse, until finally, the node features tend to become similar. Therefore, GCN architectures are very shallow at present. The road network structure is complex and the receptive field of shallow GCN is limited, so it is difficult to fully extract the spatial correlation between nodes. Consequently, when constructing the adjacency matrix, a weighted adjacency matrix was used in this study. We used Dijkstra’s shortest path algorithm to calculate the distance d(i,j) between node i and node j. The weighted adjacency matrix ${\tilde{A}}^{w}$ is defined as:

(9) $${\tilde{A}}^{w}{}_{ij}=\left\{\begin{array}{cc} d(i,j) & i\neq j\\ 1 & i=j \end{array}\right.$$

where *i* and *j* represent nodes, and the distance of the same node is d(i,i)=0. In order to retain the features of the node itself, the diagonal position is set to 1. As shown in Figure 8b, after using the weighted adjacency matrix, the one-layer graph convolution network covers the information of all nodes. The formula is:

(10) y^=Relu(A^XW)

where $\hat{y}$ is the graph convolution output, $\hat{A}={\tilde{D}}^{w}{}^{-\frac{1}{2}}{\tilde{A}}^{w}{\tilde{D}}^{w}{}^{-\frac{1}{2}}\in R^{N\times N}$ is the weighted adjacency matrix after symmetric normalization, ${\tilde{D}}^{w}{}_{ii}=\sum {}_{j}{\tilde{A}}^{w}{}_{ij}$, ReLU is the activation function, $X\in R^{N\times F\times M}$ is the input feature, F and M are the feature dimensions output after 2DTCN, and W is the weight matrix.

#### 3.2.3. Parameter Module

**Figure 9:**
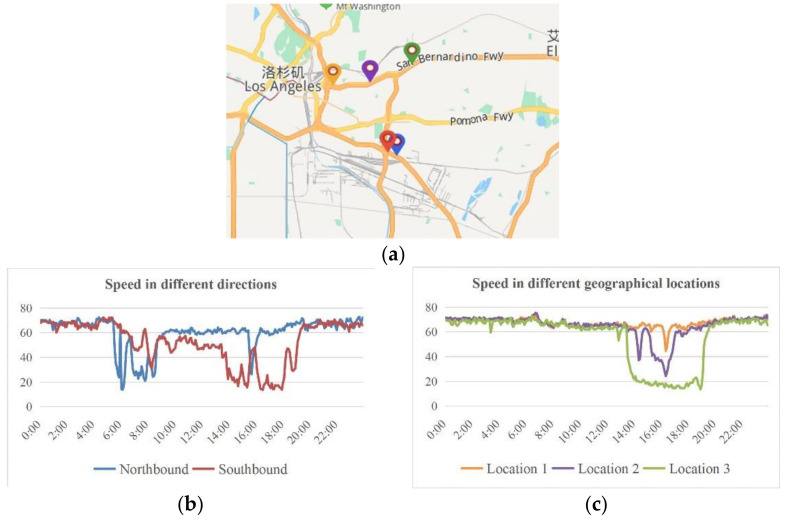
Speed comparison in different directions. (**a**) shows the location of the monitoring station. The blue mark is the northbound lane, and the red mark is the southbound lane. (**b**) shows the comparison of speed changes between two stations in different directions. (**c**) shows the comparison of speed changes between three stations located at different locations on the same road.

The geographical location, structure, grade, and direction of the road also affect the speed. As shown in Figure 9a, two sensor stations in Highway 5 and three sensor stations in Highway 10 are selected in the American PeMS system to extract their vehicle speed data for comparison. The red and blue sensor stations in Highway 5 are geographically close, but the red station is located in the southbound lane and the blue station is located in the northbound lane. Figure 9b shows the comparison of the speed changes at these two stations. Going north on the road leads to Los Angeles and, therefore, the northbound lane is seriously congested and slow moving during the morning peak hours, whereas the southbound lane is slow moving during evening peak hours. The speed change trend is related to the lane direction. The yellow, purple, and green stations are located at different locations in the same lane of Highway 10. The speed comparison results are shown in Figure 9c. The duration of the evening peak and the degree of congestion vary significantly with the location. Comparing Figure 9b,c, it can also be seen that the overall change trend of speed on different roads is different.

Table 1 shows the 10 road parameters selected for the model. The values of these 10 road parameters are fixed for each sensor and do not change with time. Therefore, it is not necessary to update the parameter matrix at any time during the rolling prediction of speed. Firstly, we normalized all parameters to form a parameter matrix $P\in R^{N\times c}$, where c=10, which is the dimensions of the road features. After dimensional transformation, the parameter matrix was spliced with $\hat{y}$, which is the output of the graph convolution module, and then the spliced data was inputted into 2DTCN to complete the extraction of the high-dimensional spatio-temporal and parameter features. Finally, the full connected neural network was used to convert the output feature dimension into N×Δ, where Δ is the number of prediction time steps.

The model uses the L2 loss function to measure the prediction performance. The loss function formula is:

(11) $$L({\hat{X}}_{t+1,}^{}\ldots ,{\hat{X}}_{t+\Delta }^{})=\sum_{i=1}^{N}\sum_{j=1}^{\Delta }({\hat{x}}_{t+j}^{i}-x_{t+j}^{i}{)}^{2}$$

where ${\hat{x}}_{t+j}^{i}$ is the predicted speed of node *i* at time *t + j*, and $x_{t+j}^{i}$ is the real speed of node *i* at time *t + j*.

## 4. Experiments and Results

### 4.1. Datasets

**Figure 10:**
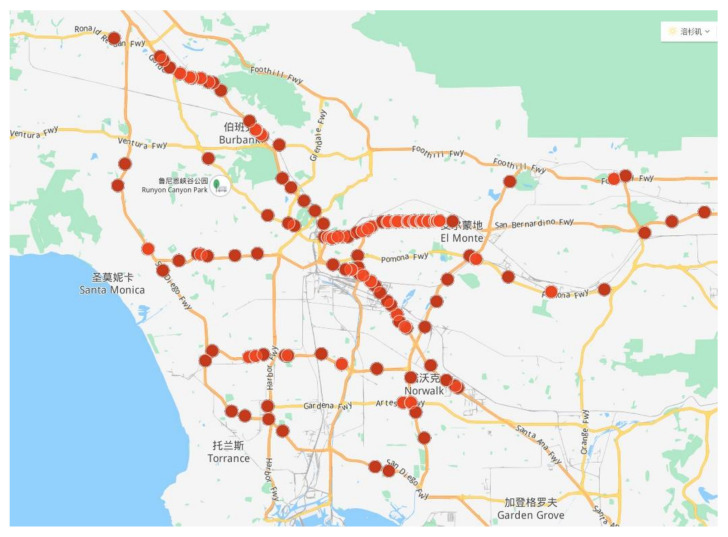
Location of the highway monitoring station.

**Figure 11:**
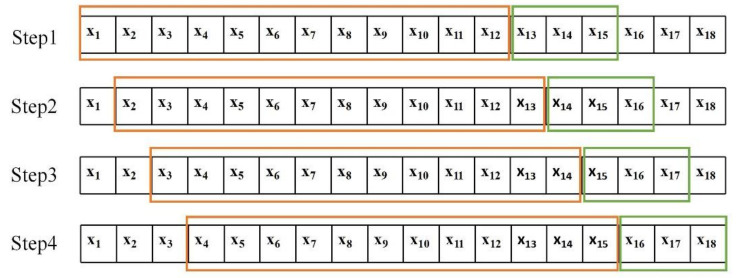
Time series data sliding window with overlapping sampling. The first step is to extract the speed data at 1–12 time points to predict the speed at 13–15 time points, the second step is to extract the speed data at 2–13 time points to predict the speed at 14–16 time points, and so on to realize rolling prediction.

The dataset used in the experiment was selected from the U.S. highway dataset PeMS. This dataset is open for download. The speed data is the average speed of the road section obtained by the sensors. It contained the data of 157 sensor stations; the locations of which are shown in Figure 10. The dataset was sampled at 5 min time intervals, and 288 slices were obtained daily for each node, with a total of 9216 slices obtained over 32 days between 1 November 2020 and 2 December 2020. The dataset was normalized by zero-mean, and 80% of the dataset was set as the training set and 20% as the verification set. In the training set, a time series sliding window with a step size of 1 was adopted to carry out overlapping sampling to create a rolling prediction of traffic speed [38]. As shown in Figure 11, the window size was 12, that is, the speed of the first 12 time slices was used to predict the speed of the next three time slices, namely, the traffic speed at 5, 10, and 15 min.

### 4.2. Experimental Parameters and Evaluation Metrics

The experimental parameter settings are shown in Table 2. When the training epoch is 100, the model output is basically stable. The learning rate of the Adam algorithm is 0.01. The greater the number of residual blocks in 2DTCN, the larger the model’s total receptive field, and the better the prediction effect. However, the training cost will also increase. Through experimental verification, when the residual block increases from one layer to five layers, MAE constantly decreases, but when the residual block is larger than five layers, the prediction effect does not change significantly, and the performance of the model tends to be saturated, but the training time is extended. Therefore, the optimal effect is achieved when the residual block is five layers. The kernels of the dilated convolution in the five layer residual blocks are all 3 × 3. The dilated rates are 1, 2, 4, 8, and 16. The padding is 2, 4, 8, 16, and 32 respectively. The output channel of FCN in 2DTCN is 64 and the output channel of GCN is 16.

In this paper, MAE (mean absolute error) and RMSE (root mean square error) were used to evaluate the error between the predicted value and the real value. The formulas of evaluation metrics are as follows:

(12) $$MAE(X_{t},{\hat{X}}_{t})=\frac{1}{n}\sum_{i=1}^{n}\left|x_{t}^{i}-{\hat{x}}_{t}^{i}\right|$$

(13) $$RMSE(X_{t},{\hat{X}}_{t})=\sqrt{\frac{1}{n}\sum_{i=1}^{n}{\left(x_{t}^{i}-{\hat{x}}_{t}^{i}\right)}^{2}}$$

where $X_{t}$ and ${\hat{X}}_{t}$ are the real value and predicted value of all nodes at time *t*, and $x_{t}^{i}$ and ${\hat{x}}_{t}^{i}$ are the real value and predicted value of node *i* at time *t*, respectively.

### 4.3. Baseline Method

In the experiment, we compared the SPTMN model with the following seven baseline models to evaluate its performance:

- LSTM [39]: LSTM is a special RNN, which can solve the problems of gradient disappearance and gradient explosion in the process of long sequence training through a gating mechanism.
- GRU [40]: Gate Recurrent Unit, which filters and processes data through updated and reset gates but has fewer parameters than LSTM.
- GCN [36]: Graph convolution neural network, which can extract the feature of graph structure through a Laplace matrix and spectral transformation.
- TGCN [27]: Temporary graph revolutionary, which combines GRU and GCN to realize traffic prediction.
- STDN [26]: Spatial-Temporal Dynamic Network, which consists of CNN and LSTM.
- STGCN [28]: Spatial-Temporal Graph Convolutional Network, which is composed of graph revolution and gated temporary revolution, and realizes the prediction of traffic flow.
- ASTGCN [30]: Attention Based Spatial-Temporal Graph Convolutional Network, which consists of GCN, standard convolution, and spatial-temporal attention mechanism.

### 4.4. Experimental Results and Analysis

In the experiment, we compared the prediction results of the SPTMN model with seven baseline models. Table 3 shows the evaluation metrics calculated from the prediction results with steps of 5, 10, and 15 min.

LSTM and GRU are basic deep learning models, which are often used to model time series. However, if they are used to model complex traffic data with many influencing factors, the data cannot be fitted by only modeling time correlation. The prediction results of these two models were the worst of all the models. STDN regards the city as an image, divides the image into regions, extracts the spatial dependence between regions by CNN, and then extracts the temporal correlation by LSTM and an attention mechanism. However, the spatial dependence of the traffic data obtained by the highway monitoring network is extremely irregular, and CNN cannot effectively extract the spatial features, so its prediction result is similar to that of GRU.

GCN can achieve feature extraction of the irregular spatial structure by spectral transformation. Generally, the prediction effect of the model is significantly improved after adding GCN. This implies that whether the topology information between traffic nodes can be sufficiently extracted has a great impact on the prediction accuracy, particularly for a sensor network with a complex structure. Compared with the STDN, the MAE of GCN decreased by 0.27, 0.29, and 0.32 over three time steps, respectively. However, GCN is insufficient to extract temporal features. To compensate for the shortcomings of GCN, TGCN adds GRU to extract time-series features based on GCN. Therefore, its evaluation metrics are higher than GCN, and its MAE is 2.70, 2.72, and 2.73, respectively, over three time steps. STGCN constitutes a temporal gated-conv block based on one-dimensional convolution and a gating mechanism, and a spatial graph-conv block based on GCN. The two blocks establish a “sandwich” structure to extract spatial-temporal correlations from different scales, so feature extraction is more efficient. Therefore, its MAE is 0.94, 1.1, and 1.1 less than that of T-GCN, respectively. ASTGCN extracts the spatial-temporal correlation of data by GCN, CNN, and an attention mechanism. In the time dimension, ASTGCN not only extracts the recent temporal features, but also fully extracts the correlation in the daily and weekly traffic flow periods. Consequently, its prediction performance is the best amongst the seven baseline models. The MAE is 1.51, 1.43, and 1.48, respectively.

SPTMN adequately considers the spatial-temporal correlation between traffic speeds and the influencing factors of road geographical structure. It uses 2DTCN and GCN to extract temporal features at different levels based on feature enhancement, extracts spatial features locally and globally, and realizes road parameter feature extraction in the parameter module. It is clear from Table 2 that the SPTMN model has the best prediction performance, with its MAE of 1.37, 1.32, and 1.37, respectively. The RMSE is 2.09, 2.01, and 2.09, respectively. Compared with ASTGCN, MAE and RMSE decreased by 8% and 8.7% on average. The experimental results adequately prove the effectiveness of the model structure design.

**Figure 12:**
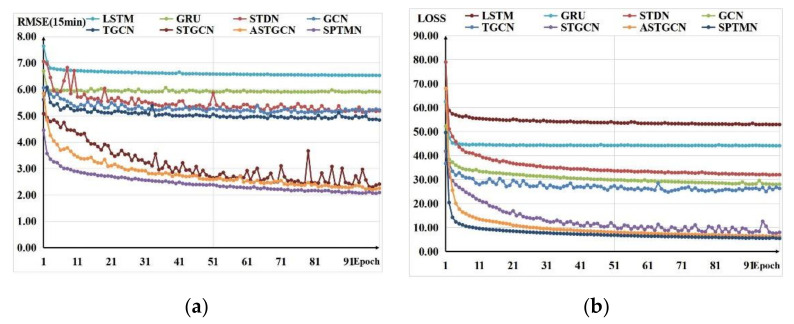
Comparison of RMSE and LOSS changes with training rounds. (**a**) shows the changes in RMSE in the 15 min prediction results. (**b**) shows the changes in the average loss value over three time steps with the epochs.

Figure 12a shows the change in RMSE in the 15 min prediction results with the training rounds. The RMSE of some models fluctuates with varying degrees during the decline, which indicates that some outliers will appear during the prediction process, resulting in a sudden increase in RMSE values. Although the RMSE of SPTMN has the fastest and most stable descent, Figure 12b shows the changes in the average loss value over three time steps with the epochs. The loss value of our proposed model decreases rapidly in the first five epochs and drops below 10 during the 10th epoch. After the 20th epoch, the decrease becomes slow, indicating that the model has converged. The above two points fully illustrate the stability and efficiency of the SPTMN model.

### 4.5. Case Study

To examine the prediction performance of the model clearly and in more detail, we selected several monitoring stations with different characteristics and compared their daily real traffic speed data with the predicted data from SPTMN, ASTGCN, STGCN, and TGCN.

**Figure 13:**
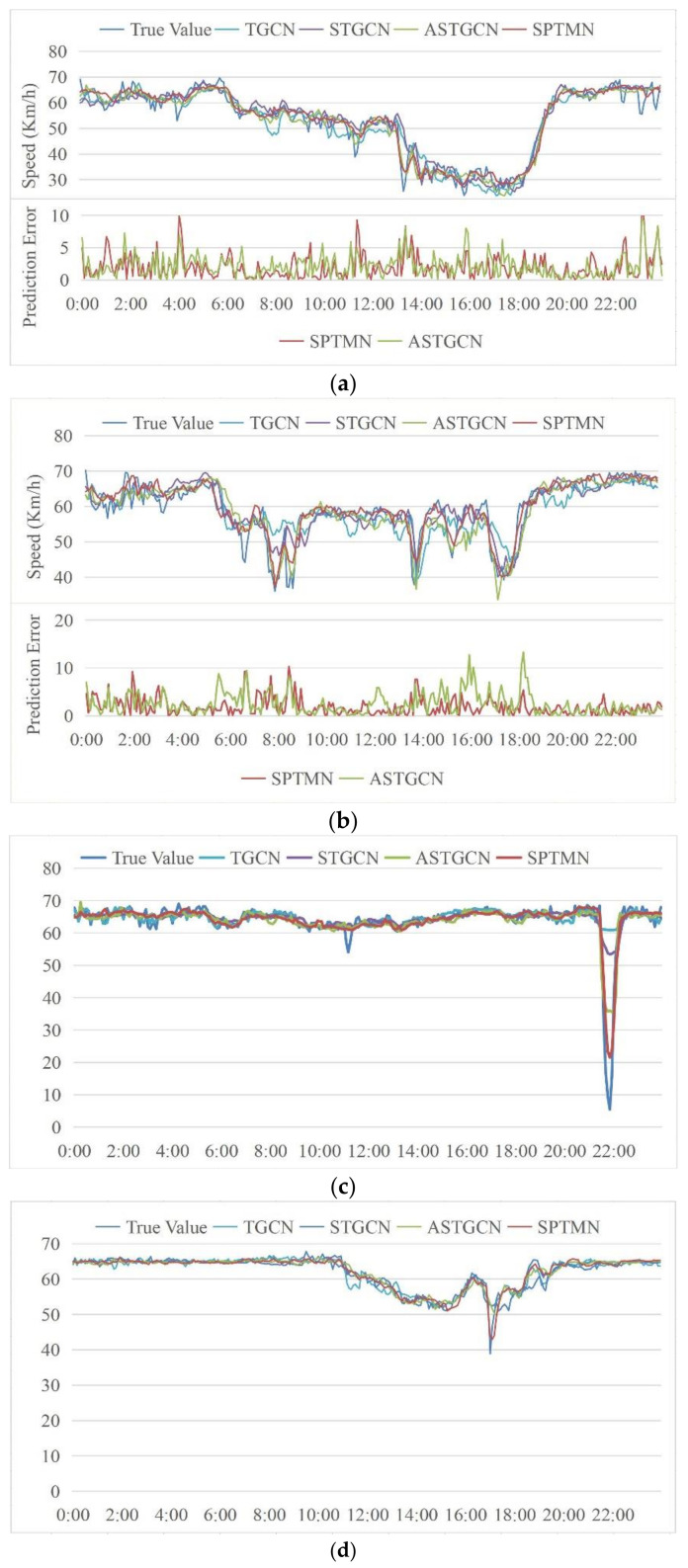
Comparison of predicted results between SPTMN and three baseline models. (**a**) The monitoring stations with a gentle speed change trend. (**b**) The monitoring stations with large and frequent speed fluctuation. (**c**) The monitoring stations with the speed drops sharply in a short time. (**d**) The monitoring stations of the HOV (High-Occupancy Vehicle) lane.

In Figure 13a,b, the upper part shows the comparison between the real data and the predicted data of each model, and the lower part shows the variation in the prediction error of SPTMN and ASTGCN models over time. The monitoring station in Figure 13a had some speed changes, but the trend is relatively flat. The prediction curve of SPTMN fits the ground truth the best. The large error is mainly concentrated in sudden changes of speed. For today’s prediction results, the MAE value of SPTMN is 1.91 and of ASTGCN is 2.16. Figure 13b shows the comparison of predicted results of each model at a monitoring station with large and frequent speed fluctuations. The prediction performance of the SPTMN model remains the most accurate. When the speed changes suddenly, SPTMN can respond quickly to the change, and its prediction error is significantly less than that of other baseline models. According to the statistics, the MAE value of SPTMN is 2.03 and of ASTGCN is 2.53. When the speed fluctuation increases, the prediction error of ASTGCN visibly increases, but the prediction error of our proposed model changes only slightly. A unique situation occurs at the monitoring station in Figure 13c, where the speed drops to close to 0 in a short time, which is a challenge for all the models. The prediction results of SPTMN fit the real data best, followed by ASTGCN, STGCN, and TGCN, which responded to this phenomenon, but the predicted speed is very different from the real data. The first three monitoring stations are all located in the Mainline. Figure 13d shows the monitoring station in the HOV lane, which is characterized by small and gradual changes in speed. All models have satisfactory prediction outcomes. At this station, the MAE of ASTGCN is 0.75, and the MAE of SPTMN is the lowest amongst all the models, at only 0.73. Overall, we conclude that our proposed model displays a more accurate and stable prediction performance under varying conditions, and therefore has a wider range of applications.

### 4.6. Ablation Study

To verify the ability of the three modules in the SPTMN model to improve prediction performance, ablation experiments were carried out on the models. This section primarily analyzes three cases, removing the feature enhancement module, GCN module, and parameter module. The experimental data and parameter settings used in this section are the same as those in the previous two sections. The quantitative results of ablation experiments are given in Table 4.

In general, the SPTMN model gives the most accurate predictive performance compared with the three variant models; specifically, the feature enhancement module has the greatest impact among the three modules. In this module, the original 2D data is expanded into 3D data through 2D causal dilated convolution to augment feature enhancement, which provides a basis for future high-dimensional feature extraction. After its removal, the prediction error increases significantly, the MAE over the three time steps increases by 1.98, 2.08, and 2.11, respectively, and the loss increases by 18.29. The impact of the GCN module is relatively small because 2DTCN also has the function of extracting the spatial dependence of data, but it cannot extract it due to the topological relationship between nodes. Therefore, the GCN module compensates for this deficiency. Without the GCN module, The MAE over the three time steps increases by 0.31, 0.66, and 0.91, respectively, and the loss increases by 0.89. Based on the first two modules, the parameter module can realize the extraction of higher-dimensional spatial-temporal features and road geographical structure features. The parameter module can decrease the MAE of the three time steps by 1.90, 1.95, and 1.93, respectively, and the loss by 24.34. Thus, the parameter module can greatly improve the prediction performance of the model. The ablation experiment demonstrates that the three modules of the SPTMN can effectively improve the prediction ability of the model, which verifies the scientific merit and effectiveness of the proposed traffic speed prediction model.

## 5. Conclusions

The evolution of traffic speed is complex and uncertain, and the prediction of traffic speed is highly challenging. In this study, a spatial-temporal graph network model SPTMN based on 2DTCN and GCN was constructed to predict short-term traffic speed. TCN is designed for time series data modeling, and traffic speed data has complex spatial correlation, in addition to time correlation. Therefore, we constructed 2DTCN based on TCN to increase the function of local spatial dimension feature extraction. In this model, we abstract the topological relationship between sensors into a graph structure, construct a weighted adjacency matrix according to the distance between nodes, and use GCN to deeply mine the global spatial correlation between nodes. Finally, other influencing factors such as road geographical location and structure are added to the model, and 2DTCN is used to extract spatio-temporal features at a higher level. The experimental results show that compared with the baseline model, the proposed model is more stable and efficient, and can adapt to the characteristics of rapid changes in traffic speed, and consequently achieves the best prediction outcomes under varying conditions.

In future research, we aim to continue to optimize the model structure and parameters, explore more complex spatial correlations, and further improve the accuracy and science behind the model. For intelligent transportation, long-term speed prediction has more enduring significance. Therefore, in future research, an important focus would be the achievement of medium- and long-term traffic flow prediction. We can also apply the SPTMN model to other spatial-temporal prediction problems, such as traffic flow prediction and travel time estimation.

## Figures and Tables

**Table 1:** Road parameters and interpretation in the SPTM model.

Parameter	Parameter Interpretation
Fwy	Highway number
Dir	The direction of the highway (N: 0, E: 1, S: 2, W: 3)
District	District where the sensor is located
County	County where the sensor is located
State_PM	The distance of a highway across a state
Abs_PM	The distance to the start of the highway
Latitude	Latitude of sensor
Longitude	Longitude of sensor
Type	Road type (ML:0; HV:1)
Lanes	Number of lanes

**Table 2:** Training parameters of prediction models.

Parameter	Parameter Value
Batch size	16
Epoch	100
Optimizer	Adam
The number of residual blocks in TCN	5

**Table 3:** Evaluation metrics of SPTMN model and seven baseline models.

Model	5 min		10 min		15 min	
	MAE	RMSE	MAE	RMSE	MAE	RMSE
LSTM	3.86	6.53	3.88	6.54	3.90	6.53
GRU	3.24	5.86	3.24	5.88	3.25	5.90
STDN	3.23	5.16	3.25	5.16	3.29	5.18
GCN	2.96	5.13	2.96	5.17	2.97	5.22
T-GCN	2.70	4.81	2.72	4.81	2.73	4.84
STGCN	1.76	2.71	1.62	2.40	1.63	2.41
ASTGCN	1.51	2.38	1.43	2.16	1.48	2.25
SPTMN	1.37	2.09	1.32	2.01	1.37	2.09

**Table 4:** Comparison of ablation study results.

Model	5 min		10 min		15 min		Loss
	MAE	RMSE	MAE	RMSE	MAE	RMSE	
SPTMN	1.39	2.12	1.33	2.02	1.36	2.07	5.42
SPTMN without feature enhancement module	3.37	5.48	3.41	5.46	3.47	5.51	23.71
SPTMN without GCN module	1.70	2.51	1.99	3.02	2.27	3.48	6.31
SPTMN without parameter module	3.29	4.99	3.28	4.99	3.29	5.02	29.76

## Data Availability

The data used in the experiment can be obtained from the following links: https://github.com/hzqhappy/SPTMN.
